# Cultures of risk and their influence on birth in rural British Columbia

**DOI:** 10.1186/1471-2296-13-108

**Published:** 2012-11-16

**Authors:** Jude Kornelsen, Stefan Grzybowski

**Affiliations:** 1 Centre for Rural Health Research, Department of Family Practice, University of British Columbia, Vancouver, Canada

**Keywords:** Access to care, Rural and remote, Maternity care, Canada, Risk perception

## Abstract

**Background:**

A significant number of Canadian rural communities offer local maternity services in the absence of caesarean section back-up to parturient residents. These communities are witnessing a high outflow of women leaving to give birth in larger centres to ensure immediate access to the procedure. A minority of women choose to stay in their home communities to give birth in the absence of such access. In this instance, decision-making criteria and conceptions of risk between physicians and parturient women may not align due to the privileging of different risk factors.

**Methods:**

In-depth qualitative interviews and focus groups with 27 care providers and 43 women from 3 rural communities in B.C.

**Results:**

When birth was planned locally, physicians expressed an awareness and acceptance of the clinical risk incurred. Likewise, when birth was planned outside the local community, most parturient women expressed an awareness and acceptance of the social risk incurred due to leaving the community.

**Conclusions:**

The tensions created by these contrasting approaches relate to underlying values and beliefs. As such, an awareness can address the impasse and work to provide a resolution to the competing prioritizations of risk.

## Background

There has been a sudden decline in the number of rural communities across Canada offering local maternity care since 2000 [1-3] due to a confluence of factors including the regionalization of health services delivery in many jurisdictions [4], physician recruitment and retention challenges [5], limited access to midwives [6,7], and diminished access to nurses trained in obstetrics [8]. A significant number of communities that continue to offer local maternity services to parturient residents in the absence of surgical back-up are witnessing a high outflow of women leaving to give birth in larger centres in order to ensure immediate access to caesarean section capabilities. This type of outflow is not unique to Canada and has also been observed in other medically advanced countries such as the United States and the United Kingdom [9,10]. A minority of women from these communities choose to stay in their home communities to give birth [11].

An overview of the rural maternity care research literature suggests that the onus has been to prove the safety of small rural maternity care services in the face of assumptions that they were less safe than centralized, specialized service units. Most studies focusing on the safety of rural maternity care services have used perinatal mortality rates and birth weight-specific perinatal mortality rates as the key outcome measure [12]. By comparing Level I (general practitioners only) facilities’ birth outcomes with those of Level II (regional referral hospital staffed by specialist obstetricians) and Level III (highly-specialized obstetric and neonatal care) facilities, researchers have sought to uncover whether or not Level I services are equally or less safe than more specialized obstetric centres [13-26]. When taken together, the evidence from this body of research supports the assertion that “‘safety cannot be used as a basis for centralizing birth care in large Level III facilities” [27]. Furthermore, the question of whether or not rural maternity care services require caesarean section capabilities is not definitively addressed in the literature, despite the number of rural obstetric units that, historically, have operated without local surgical backup [13,16,26-28].

Most importantly, the literature has not systematically explored the implications of *no* local access to maternity services in rural communities. In a pioneering study, Larimore and Davis showed that in rural Florida counties there was a negative correlation between availability of maternity care services and infant mortality (*R = -0.42, P = .012*) [29]. Among the speculated negative social consequences of losing local maternity care services are the potentially harmful stressors associated with pregnant women traveling for perinatal care [2,30]. The implications of barriers to access to local care are exacerbated for populations with less material and social resources [31,32].

The decision to offer local maternity services in the absence of caesarean section capability is a complex one requiring the alignment of administrators and care providers, the support of health authorities, and the expressed desire of community members within a realistic understanding of risks incurred. Beyond these structural influences, individual practitioners must assess the candidacy of each individual woman who wishes to stay in the community. This is usually done within a context of risk assessment. Contemporary risk assessment combines the clinical judgement of care providers with policy guidelines and standardized risk assessment indices [33,34]. As perinatal services have been regionalized, standardized risk-scoring systems have become increasingly popular as a way to identify ‘high-risk’ pregnancies that need the services found in larger, regional centres [33,35,36], despite challenges to the accuracy and efficacy of such indices [37-39]. At issue, however, is the privileging of quantifiable measures to the exclusion of those unquantifiable factors that have profound influence on the nature of pregnancy, labour, and delivery [33,34,40]. It has further been suggested that the excessive reliance on risk assessment tools may give rise to a reductionist view of pregnancy and birth as a biomedical event as opposed to a holistic and integrated life process [34,39,41].

The decision of whether a woman stays in her local community to birth or leaves prior to the onset of labour to birth in a referral facility is not made by the care provider alone, but in ideal situations is a product of a process of shared decision-making between a care provider and patient. As argued by Mackenzie-Bryers and Teijlingen, a shared decision making model allows the experiences of the patient to be incorporated into the decision-making process [10]. To date, while it is being acknowledged that there are differing views of what constitutes risk between clinicians and parturient women [10,42], there remains a need to understand what factors rural parturient women weigh when assessing risk. Particular attention needs to be paid to whether or not women’s views reconcile with care providers’ priorities. This exploratory, qualitative investigation sought to answer the question, “What are the maternity care experiences of rural care providers and parturient women including their perspectives on risk?” We will consider rural women and care providers’ perspectives on risk, acknowledging the frequent eclipse of one by the other due to the lack of understanding of each other’s priorities.

## Methods

Open-ended interviews and focus groups were undertaken in three rural communities in British Columbia chosen to represent diversity in population ethnicity and culture, geography (including distance to referral centre with caesarean section capability), and usual weather conditions (see Table 1). All primary care providers in the three communities were recruited by mail and follow-up phone calls to participate in interviews; women were recruited using a third-party recruitment process through local public health nurses and through the “snowball technique.” Inclusion criteria for women included being a resident of the study community and having given birth in the past 2 years. Care providers were required to be practicing and living in the study community and to be offering maternity care as part of the practice.

**Table 1:** Community background

	**Community 1**	**Community 2**	**Community 3**
Yearly Weather Conditions^a^	Temp (Summer): 5° to 20°C	Temp (Summer): 18°C	Temp (Summer): 19°C
	Temp (Winter): 5° to -15°C	Temp (Winter): 4°C	Temp (Winter): 2°C
	Precipitation:2500 mm	Precipitation: 7559 mm	Precipitation: 6284 mm
	Snowfall: High levels in the winter months	Snow: Rare	Snow: Occasional snowfall in winter months
Travel Distance Time to Referral Centre^b^	452 km	203 km	298 km
	6 h travel over land	5 h travel via car and ferry	7 h travel via car and ferry
Demographics^c^	Population: 135	Population: 1045	Population: 940
	Catchment: 2897	Catchment: 3000	Catchment: 2500
	50% Aboriginal	37% Aboriginal	37% Aboriginal

*Source:* Data adapted from http://rccbc.ca*.*

Notes:

^a^“Temp” refers to the average seasonal temperature. “Precipitation” represents the annual average rainfall in the community.

^b^Travel time reflects the average length of time during optimal weather conditions that it takes to access the nearest designated labour and delivery service with caesarean section back-up.

^c^“Catchment” refers to the population living within one-hour travel time of the local hospital. This includes people who live in smaller, surrounding communities. “Aboriginal” people are the original peoples of Canada and include First Nations, Inuit, and Metis groups. We have included the percentage of Aboriginal people living in the one-hour catchment.

Interviews were conducted by the co-investigators (JK, SG) and assisted by a research coordinator and two research assistants. The co-investigators are a health services researcher (JK), who has studied the social dimensions of childbirth, and family physician researcher (SG), who worked as a rural family physician providing maternity care in an isolated rural community for 11 years. In each interview, one team member conducted the interview and one member took notes. Each interview lasted between 30 and 90 minutes and took place in the participant’s home community in a location determined by the participant (primarily in a local health centre, hospital, or coffee shop). All interviews were audio recorded with the participants’ permission and transcribed.

Thematic analysis of transcripts and field notes was undertaken by the co-investigators and the research coordinator through the following steps: (1) transcripts and field notes were read and re-read until team members had a thorough understanding of the data; (2) initial coding for themes was undertaken independently on eight transcripts and independent code books were constructed by one of the co-investigators (JK) and the research coordinator; (3) code books were compared to determine level of congruence; (4) a composite code book was created by one of the co-investigators (JK) to guide coding of the complete data. The primary framework for analysis was a logic model framework including articulation of activities, resources, outcomes, outputs, and impacts. A secondary, open analysis of the data was undertaken by the co-investigators and yielded the themes of safety and risk [43,44]. Preliminary findings were presented to participants and other members of the communities, and participants expressed a high level of resonance between the findings and their experiences. This paper will focus on the theme of risk and safety. Ethical approval for this research was sought and received from the University of British Columbia’s Behavioural Research Ethics Board.

## Results

Interviews were conducted with a total of 27 care providers and 43 women (9 of whom gave birth in their home community and 34 of whom delivered away). The care providers included general practitioners (GPs), labour and delivery nurses, operating room nurses, one GP Surgeon, and one GP Anaesthetist. In the community with the GP Surgical service, local caesarean section back up was available only intermittently. The annual number of deliveries for each community was small, with fewer than 35 annual births in each community to women living within one hour of the local hospital. In all three communities, including the community with a GP Surgical service, there was a high outflow of birthing women to referral centres for labour and delivery services.

Perspectives on risks differed between physicians and birthing women: the *immediacy* of the stress associated with risk depended on the place of birth. When birth took place in the home community, nurses and physicians expressed an awareness of clinical concerns about the management of labour, and fears about the potential for a bad outcome. When birth was away, women expressed their experience of the significant anxiety that social stressors cause, due to separation from family and community. These disparate concerns reflected participants’ underlying values (see Table 2).

**Table 2:** Women and care providers’ views on social and clinical risk

**Clinical Risk of Staying**	“It is more stressful with primips. And I’ve been in the plane with two or in the helicopter overnight with two primips in the past couple of years going [to the referral community] in labour [with] stuck babies and you know it’s, it is stressful and eventually I can see … it leading to me being a lot more reluctant.” (Care Provider 1, Community 1)
	“Yeah and now it’s like you know they send people off regardless and at one time I had kind of a negative attitude about that but after having seen [it] you never know when things could go really, really, really wrong and when people are stranded here with the weather. Like, there are lots of times when nothing moves. And yeah like it would be really wonderful if everybody could have their babies at home or in the hospital here but as far as safety goes, I’m not sure if that’s really an option.” (Care Provider 1, Community 1)
**Social Risk of Leaving**	“I would just sort of like to not even think about leaving and just [stay] at home. I would almost rather risk that kind of a trip than have to be away from home for so long.” (Participant 12, Community 2)
	“[When] you have the same doctor they know what’s going on with your body … I think it would … be very uncomfortable for a first time mum to have to have seven different doctors.” (Participant 1, Community 3)
	“And it’s the poorer women that sort of suffer the most. Because you know then they’re having to make the choice to stay up here. Well it’s not a choice for them. They’re having to stay up here and then, you know, having to risk the mad dash [out of the community] just because they don’t have the money to stay there. And they’re away from all their support.” (Participant 12, Community 2)
	“Cause I’m a single mother and I’ve got four kids, I mean that’s a scary thing for me, and then I had to get respite set up in case I went into the hospital and I didn’t have anybody around and that was a scary thought too because then that’s getting the Ministry involved.” (Participant 7, Community 3)
	“We were having children in our homes [and] we were having children naturally long before doctors came to be. We were having children just the way I had my child eight months ago.” (Participant 6, Community 2)

### Clinical risk

All care providers in this study expressed a high level of awareness of the clinical risks involved in offering maternity care in communities without local surgical back-up, underscored by an acknowledgment of the unpredictability of birth. These risks were perceived to increase with a problematic reproductive history, compromised health or social status, and by parity, with nulliparous women being perceived to have the highest risk. Weather concerns, leading to challenges in transport out of the community, accentuated perceptions of clinical risk for almost all providers.

Care providers also expressed an awareness of the clinical risks involved in an unplanned precipitous delivery in the community. This might occur due to the onset of labour prior to the time of relocation to the referral community, or the “10 cm strategy,” which indicates that a woman who is dilated 10 cm or more can no longer be be safely transferred [45]. This led to strategies to minimize the potential for “high risk” women going into labour in the communities. Strategies include developing an awareness of risk factors for preterm labour and a sense of acuity in predicting who may either elect to stay beyond the recommended time or who may travel to the referral community for a short time but return before the birth.

Additionally, care providers also recognized their own personal social risks of practicing within a low-technology context which included the social stigma in the case of a bad outcome. This was predicated on their experiences of the integrated, multiple relationships in rural communities and the wide web of associational ties they had with community members. Several participants said that this vulnerability could be mitigated by a community process that demonstrated a realistic understanding of the risks of local care without surgical back-up and a willingness to accept these risks.

### Social risk

For women who chose to deliver in a referral centre, social risks were paramount and included: leaving families and support systems behind, the lack of continuity of caregiver, loss of positive attributes of birthing in the community, and financial challenges incurred by leaving. Leaving families and support systems, and the lack of social support this engendered were highlighted by almost all of the participants. For those who were able to access prenatal care in their community, leaving the community also meant a disruption in their relationship with their care provider. The realities of financial stressors were also perceived as a risk to leaving the community, particularly in instances when the additional costs of travel and accommodation were prohibitive for the evacuating family.

Many of the participants, particularly Aboriginal women, acknowledged the cultural risk to birthing outside the community and a perceived illogic to the precipitous change in historical practices. Several spoke of the sadness in not having their community name on their child’s birth certificate and others recalled the long history of practice of birth in the community.

Participants in this study revealed thematically consistent ways in which they mitigated their perceived risks of leaving the community. One common response was to secure social support in the referral community, either by bringing family and friends or selecting a community in which to birth based on the presence of an existing support network (usually extended family). The importance of securing access to additional financial support was also noted, primarily by Aboriginal women, who had access to funding through their bands for travel, accommodation, and, in some instances, escort accompaniment to the referral community. Others noted additional support sought through the appropriate government agencies, although not without concern about the implications of engaging in ‘the system.’

## Discussion and conclusions

The question of “risk” in medicine has traditionally prioritized a clinical perspective as defined by care providers. This approach has excluded adequate consideration of the psycho-social influences that have a substantial impact on patients’ decision-making processes and disregards our growing understanding of health as a state of physical, mental, and social well-being [43]. Not surprisingly, care providers and patients may value different components of health, which will give rise to different interpretations of risk. This is acutely evident in maternity care where there is continued dissonance in attitudes toward interventions in birth [46,47] and protocols such as induction for post-dates pregnancies [47]. In a rural environment with limited access to caesarean section services, the different values placed on the physical/mental/social dimensions of health have frequently led to a privileging of the medically defined course of care. Women in this study identified social priorities in their decision for location of birth that were not adequately addressed by care providers. The implications of this are significant and can lead to an antagonistic relationship where neither the care provider nor woman feels heard. This can result in care providers viewing parturient women as non-compliant and women feeling abandoned, and ultimately manifests in increasingly stringent definitions of “high risk” pregnancy and inappropriate referral out of the community. In extreme circumstances, women may choose unassisted home births or arrive at a local hospital in advanced labour to preclude transfer to a referral community. Parturient women who engage in such activities to mitigate the social risks of leaving their community face increased clinical risks leading to potentially adverse outcomes. Findings from Grzybowski, Stoll, and Kornelsen indicate that women who live 1-2 hours away from labour and delivery services have an increased risk of an unplanned out-of-hospital delivery [48]. This study also illustrates that women who live 2–4 hours from services have increased rates of intervention including the highest rates of induction of labour for logistical reasons (i.e. to electively induce labour so that they may return to their home community sooner). Further, newborns of mothers who live more than 4 hours from services are at increased risk of perinatal mortality (adjusted OR 3.17, 95% CI 1.45–6.95) [48]. Ironically, providers tend to interpret the potential for adverse outcomes as cause for encouraging women to deliver in referral and tertiary communities, a strategy that may increase the social stress women experience and be associated with the complications that care providers are trying to avoid. Conversely, if birthing women remain in the community, rural care providers may incur stress due to the uncertainty of providing intrapartum care and the potential for community backlash if a bad clinical outcome occurs. The social risks for these practitioners can be significant enough for some to choose to cease providing intrapartum care or leave their community altogether [49].

Ultimately, the concept of risk is applied to minimize adverse outcomes for a population [50]. For rural parturient populations, there are crucial nuances to our understanding of risk that include parity, degree of isolation, and vulnerability of the population. For instance, in nulliparous pregnancies where there is a heightened risk for surgical intervention in comparison to multiparous pregnancies, care providers can be uncomfortable offering local delivery and encourage nulliparous women to deliver away from their home community. Multiparous patients, on the other hand, have potentially higher social risks, as the responsibility of caring for their previous children compounds the complexity and costs of delivering away. Multiparous patients are also at higher risk of precipitate delivery (low risk for delivery locally but at high risk for delivery en route if delivering away and traveling in early labour) [51]. Geographic isolation is a key consideration in any decision surrounding a rural woman’s planned birth. Is weather a factor? What are the risks of delivery en route if traveling to a referral site in early labour? What is the risk of intrapartum transfer? Furthermore, the overall vulnerability of the population needs to be considered. If aggregate socio-economic status, educational background, and health status is low, how likely are ‘unexpected’ complications? How important are the mitigating influences of culture and family?

A balanced approach to risk management grounded in a comprehensive approach to health is a necessary step towards better health services for rural parturient women and their babies. This approach will need to weigh the issues care providers and women consider in their risk assessment strategies. An efficacious way to do this may involve development and testing of risk discussion guides and decision-making tools. Such tools may augment the communicative process by making explicit the values and concerns guiding each perspective.

### Limitations of study

The relationship between geographic realities and access to specialists in referral centres dominates the debate on safety and risk in rural maternity care. Although selection criteria for community study sites included diversity of geographic circumstances, including distance and conditions of access to the nearest referral centre, the geographic diversity of rural communities cannot be represented by three study sites. Caution must thus be exercised in transferring findings to dissimilar geographic locations.

Although Aboriginal women were included in this study, they were not specifically recruited nor was the research undertaken within any Aboriginal communities. We recognized differences in experiences between Aboriginal and non-Aboriginal participants early in the data-gathering process, emanating from the strength of kinship ties in many Aboriginal communities and the subsequent importance of extended family during labour, delivery, and the postpartum period. Additional differences included socially complex factors such as the lower age for childbearing for Aboriginal women in Canada [52], as well as higher rates of substance abuse and medical conditions that may complicate pregnancy [53-55]. These differences suggest that nuances of experiences may not be transferable to rural Aboriginal women’s experiences of birth. The need for research on this particular demographic is being answered, as seen in the recent study published by Driscoll and Kelly [56].

## Competing interests

The authors declare that they have no competing interests where appropriate.

## Authors’ contributions

JK contributed to conceptualizing the study, implementation and data analysis and was the lead writer of the manuscript. SG contributed to conceptualizing the study, implementation and the writing of the manuscript. Both authors read and approved the final manuscript.

## Pre-publication history

The pre-publication history for this paper can be accessed here:

http://www.biomedcentral.com/1471-2296/13/108/prepub
